# Physical Activity Predicts Performance in an Unpracticed Bimanual Coordination Task

**DOI:** 10.3389/fpsyg.2017.00249

**Published:** 2017-02-20

**Authors:** Matthieu P. Boisgontier, Leen Serbruyns, Stephan P. Swinnen

**Affiliations:** Department of Kinesiology, Movement Control and Neuroplasticity Research Group, Group of Biomedical SciencesKU Leuven, Leuven, Belgium

**Keywords:** bimanual coordination, computer games, health, music, physical activity

## Abstract

Practice of a given physical activity is known to improve the motor skills related to this activity. However, whether unrelated skills are also improved is still unclear. To test the impact of physical activity on an unpracticed motor task, 26 young adults completed the international physical activity questionnaire and performed a bimanual coordination task they had never practiced before. Results showed that higher total physical activity predicted higher performance in the bimanual task, controlling for multiple factors such as age, physical inactivity, music practice, and computer games practice. Linear mixed models allowed this effect of physical activity to be generalized to a large population of bimanual coordination conditions. This finding runs counter to the notion that generalized motor abilities do not exist and supports the existence of a “learning to learn” skill that could be improved through physical activity and that impacts performance in tasks that are not necessarily related to the practiced activity.

## Introduction

**FIGURE 1:**
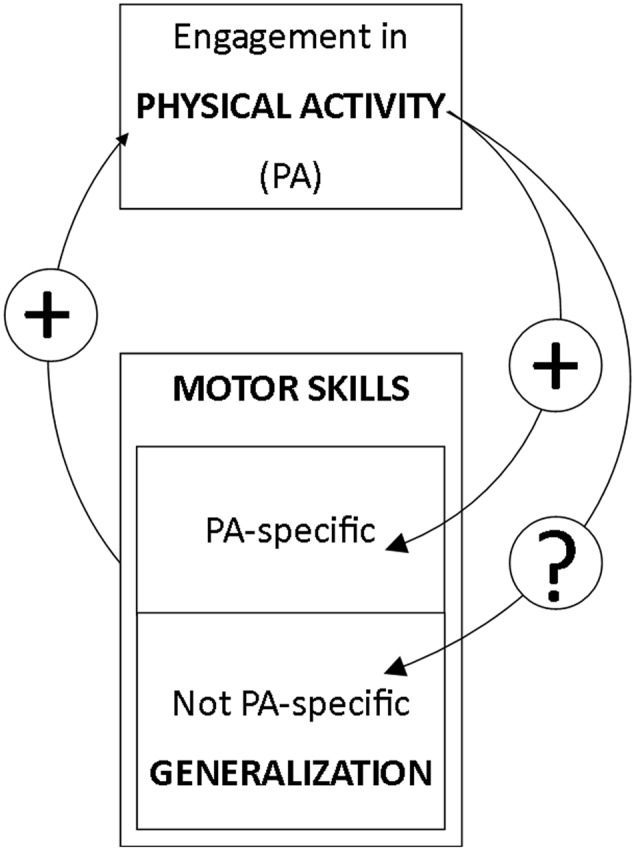
**Scope of the study.** Here we posit that physical activity engagement and motor skill proficiency can potentially reinforce each other via a virtuous circle. We further hypothesize that physical activity (PA) is associated with a global skill improvement that is likely related to the “learning how to learn” phenomenon (Harlow, 1949; Seidler, 2010), which is expressed in skills specific to the practiced PA, but also to other skills, not specific to the practiced PA.

Each year, physical inactivity is responsible for 13 million of disability-adjusted life-years worldwide and costs 67.5 billion of international dollars (Ding et al., 2016). In this context, the promotion of physical activity appears strongly relevant. Such promotion could be initiated through the development of motor skills that have shown to be a primary factor of engagement in physical activity (Nougier et al., 1996; Stodden et al., 2008; Barnett et al., 2009). While engagement in a certain type of physical activity is known to improve the motor skills specific to this activity (Bachman, 1961; Higbie et al., 1996), it is still unclear whether movement skills that are not directly related to this activity would also be improved. If such a generalization does occur, engagement in one type of physical activity could potentially promote engagement in other activities (**Figure 1**).

A potential explanatory mechanism of such overall improvement is the “learning to learn” phenomenon (Harlow, 1949; Seidler, 2010), whereby problem-solving skills developed for one task/context generalize to similar tasks/contexts (Bock et al., 2001; Seidler, 2004). This generalization could be explained by adaptations at the level of executive functions (e.g., attention, response planning, decision making) that have been linked to physical activity (Kraft, 2012), and/or by improved proprioception (Adamo et al., 2009; Helsen et al., 2016), which has shown to be critical for the control of movement (Sainburg et al., 1995; Nougier et al., 1996; Jackson et al., 2000). Both improved proprioception and an increased ability to reduce limb stiffness, which would allow movements with larger amplitudes, would result in larger perceived errors to learn from (Burdet et al., 2001). Finally, as suggested by Seidler (2010), this generalization could be explained by the improvement of the core skills required for the learning process, such as pattern and error detection and correction.

Here, we hypothesized that physical activity predicts motor skill proficiency in tasks that have never been practiced before such as a multi-frequency bimanual coordination task. We further posited that physical activity engagement and motor skill proficiency can potentially reinforce each other via a virtuous circle (**Figure 1**).

## Materials and Methods

### Participants

Twenty-six healthy young volunteers (age range 18–30 years; mean age 24 ± 3 years) participated in the study. All participants were right-handed according to the Edinburgh Handedness Inventory (Oldfield, 1971). The International Physical Activity Questionnaire (IPAQ; Craig et al., 2003) was used to assess total physical activity and inactivity. Participants were asked how much time per week they spend sitting (physical inactivity), play a music instrument, and play computer games. All participants had normal or corrected-to-normal vision, and none reported neurological, psychiatric, or cardiovascular disorders. This study was carried out in accordance with the recommendations of the guidelines established by the ethics committee for biomedical research at KU Leuven. All subjects gave written informed consent in accordance with the Declaration of Helsinki. The protocol was approved by the KU Leuven, Belgium.

### Physical Activity

Total physical activity was assessed using the IPAQ, which assesses physical activity undertaken across a comprehensive set of domains including leisure time, domestic and gardening activities, and work-related and transport-related activities. The specific types of activity are walking, moderate-intensity activities, and vigorous-intensity activities. Frequency (days per week) and duration (time per day) are collected separately for each specific type of activity. The total score used to describe total physical activity required weighted summation of the duration (in minutes) and frequency (days) of walking, moderate-intensity, and vigorous-intensity activity. Each type of activity was weighted by its energy requirements defined in Metabolic Equivalent of Task (METs): 3.3 METs for walking, 4.0 METs for moderate physical activity, and 8.0 METs for vigorous physical activity (Ainsworth et al., 2000).

### Experimental Setup

Skilled movement proficiency was assessed using a bimanual tracking task (Sisti et al., 2011; Beets et al., 2015; Serbruyns et al., 2015; Maes et al., 2017). Participants were seated in front of a computer monitor with the forearms resting on a custom-made adjustable ramp. A 5-cm diameter dial was mounted at the end of each ramp. The task consisted in turning the handle of the dials with the thumb and index according to specific patterns without visual feedback of the upper limbs. Angular displacements were registered by means of non-ferromagnetic optical shaft encoders (Avago Technologies, sampling frequency = 100 Hz, accuracy = 0.089°) fixed to the rotation axes of the dials. The gain was set to 10 arbitrary units (au) per rotation indicating that drawing a vertical or horizontal line on a screen consisting of 162 au, required 16.2 complete rotations of the left or right dial, respectively. Rotating the dials moved a red cursor on the screen serving as online visual feedback.

### Bimanual Tracking Task

**FIGURE 2:**
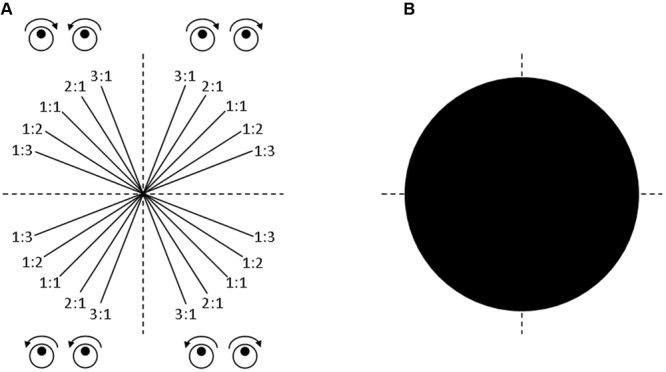
**Bimanual coordination task.** Illustration of the 20 bimanual coordination conditions **(A)**. Considering these conditions as random in a linear mixed model allows the results to be generalized to all the possible conditions **(B)**.

Participants were instructed to track a white target dot moving along a target line by rotating both dials simultaneously. Four coordination patterns imposed by the target line direction were tested: both hands rotating inward, outward, clockwise, or counter-clockwise. The left and right hands, respectively, controlled movements on the vertical and horizontal axis. Each pattern was performed according to five frequency ratios: 1:1, 1:2, 1:3, 2:1, and 3:1 (left hand:right hand) resulting in 20 different target line directions (**Figure 2A**).

### Procedure

Prior to data recording, participants performed 12 trials with different target lines for familiarization. Before each recorded trial, the target line appeared for 2 s. Then the target dot moved over the line at a constant speed from start (center of the screen) to end for 10 s. The goal was to match the white target dot movement with the red dot as accurately as possible in both space and time. The inter-trial interval was 3 s. Four 6-min blocks with 3 min rest in between were administered, each consisting of 24 trials, presented in a pseudorandom order. Therefore, all participants performed 96 recorded trials. Each block included all 20 distinctive target lines and an additional 1:1 trial for each coordination pattern.

### Kinematic Data Analysis

Accuracy was assessed using the target deviation of the time series and was computed as follows for each trial:

$$\begin{array}{c} Target\;Deviation=\sum_{1}^{n}\sqrt{{\left(x_{2}-x_{1}\right)}^{2}+{\left(y_{2}-y_{1}\right)}^{2}} \end{array}$$

where *n* is the number of data samples over a trial of 10 s (10 × 10^2^), x_2_ and y_2_ are the respective position of the red cursor on the *x-* and *y* axis, and x_1_ and y_1_ are the respective position of the white target dot on the *x-* and *y* axis. Larger target deviation scores reflected poorer performance.

### Statistical Analysis

The extent to which total physical activity, music practice, and computer gaming were predictive of target deviation was analyzed using a linear mixed model. Unlike traditional analyses of variance, linear mixed models take into account the sampling variability of both the participants and conditions, thereby limiting a large inflation of false positives (Boisgontier and Cheval, 2016). Moreover, as illustrated in **Figure 2B**, treating both participants and conditions as random effects allows generalizing the results to the population of participants, but also to the population of conditions (Barr et al., 2013). Finally, linear mixed models take each single observation into account, thereby avoiding information loss due to averaging. The linear mixed model specified participants (*n* = 26) and target lines (*n* = 20) as random factors and was built using the R language lmerTest package, version 2.0-30^1^. Target deviation was normalized using the Box-Cox method. The predictive variable was total physical activity as measured by the IPAQ. The control variables were age, gender, session, trial order, physical inactivity, computer game practice, and music practice. The continuous variables were scaled and centered on zero.

## Results

**FIGURE 3:**
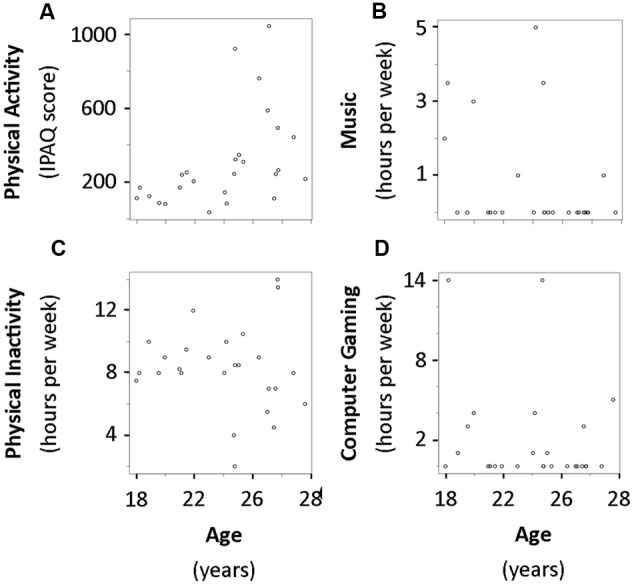
**Raw data of PA (A)**, music practice **(B)**, physical inactivity **(C)**, and computer game practice **(D)** as a function of age. IPAQ, International Physical Activity Questionnaire.

The distribution of physical activity, music practice, computer game practice, and physical inactivity are illustrated as a function of age in **Figure 3**.

**FIGURE 4:**
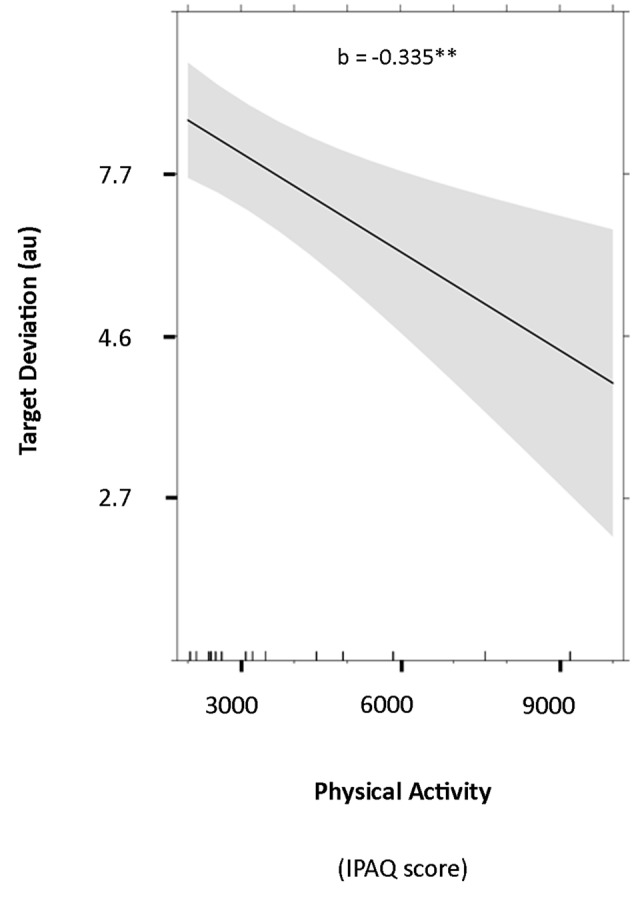
**Fixed effect and the 95% confidence interval of total PA on target deviation.** This effect was significant while controlling for all the other factors reported in **Table 1**. The scale of target deviation was back transformed. IPAQ, International Physical Activity Questionnaire. ^∗∗^*p* < 0.01.

Results of the linear mixed model (**Table 1**) showed a significant effect of physical activity (*b* = -0.335, *p* = 0.005) on target deviation with higher total physical activity predicting lower target deviation and thus better performance (**Figure 4**). This effect was observed while controlling for age (*b* = 0.142, *p* = 0.101), gender (*b* = -0.241, *p* = 0.178), session (*b* = -0.160, *p* < 0.001), trial order (*b* = -0.001, *p* = 0.660), physical inactivity (*b* = -0.140, *p* = 0.137), computer game practice (*b* = -0.021, *p* = 0.863), and music practice (*b* = -0.286, *p* = 0.009). These latter results showed that the number of hours of music practice per week, but not computer games practice, predicted lower target deviation.

**Table 1:** Predictors of target deviation.

Fixed effects	*b*	*SE*	*p*
Intercept	2.499 × 10^0^	1.200 × 10^-1^	<0.001^∗∗∗^
Age	1.417 × 10^-1^	8.337 × 10^-2^	0.101
Gender	-2.408 × 10^-1^	1.739 × 10^-1^	0.178
Session (1–4)	-1.601 × 10^-1^	1.069 × 10^-2^	<0.001^∗∗∗^
Trial order (1–24)	-7.646 × 10^-4^	1.737 × 10^-3^	0.660
Physical inactivity	-1.398 × 10^-1^	9.097 × 10^-2^	0.137
Computer games	-2.078 × 10^-2^	1.197 × 10^-1^	0.863
Music	-2.860 × 10^-1^	1.008 × 10^-1^	0.009ˆ**
Physical activity	-3.349 × 10^-1^	1.082 × 10^-1^	0.005ˆ**
**Random effects**	**σ^2^**		
Participant			
Intercept	1.187 × 10^-1^		
Condition			
Intercept	4.550 **×** 10^-2^		
Residual		3.567 × 10^-1^	

*^∗∗^*p* < 0.01, ^∗∗∗^*p* < 0.001.*

## Discussion

Here we investigated whether engagement in physical activity improves overall motor skill proficiency by means of an unpracticed bimanual coordination task. Results revealed that usual physical activity was predictive of performance in multiple conditions of this new and complex bimanual coordination task. This result is consistent with studies showing a relationship between the level of physical activity and motor speed. Specifically, in a discrete unimanual aiming task young active adults showed faster reaction times and movement times than sedentary young adults, while accuracy was similar (Van Halewyck et al., 2014; Helsen et al., 2016). Reaction times were also faster in active young and older adults than their sedentary peers (Lupinacci et al., 1993).

Our results demonstrate that higher total physical activity enhances performance on an unpracticed task. This result supports previous findings showing transfer between unrelated skills of a unimanual joystick tracking (Bock et al., 2001) or aiming task (Seidler, 2004) and reveals that such transfer can be demonstrated using a metric encompassing multiple skills (i.e., total physical activity). These findings run counter to the notion that motor abilities are highly specific and that practice effects are skill and context specific (e.g., Higbie et al., 1996). Conversely, our findings support the existence of a “learning to learn” skill (Harlow, 1949; Seidler, 2010) that could be improved through physical activity and that would impact performance in tasks that are not necessarily related to the practiced activity. Future studies should investigate, whether this impact of physical activity is related to the participants’ experience with a wide range of activities or to their overall level of fitness. Here, we showed that this impact was significant, irrespective of music practice, and computer gaming. Future research should also follow up on the significant relation between music practice and bimanual coordination to see if this relation is mediated by different processes or mechanisms other than those that underlie the relation between total physical activity and bimanual coordination.

Finally, as motor skills are known to be a primary factor for engagement in physical activity, these results are encouraging for the promotion of physical activity. Indeed, they reveal that once engaged in physical activity, this engagement could potentially be auto-reinforced through an overall improvement of motor skill proficiency.

## Author Contributions

Experimental design: SS and LS. Experimental conduct: LS. Data analysis: MB. First draft preparation: MB. Manuscript preparation: MB and SS.

## Conflict of Interest Statement

The authors declare that the research was conducted in the absence of any commercial or financial relationships that could be construed as a potential conflict of interest.

## Footnotes

1 http://www.r-project.org/
